# Development and validation of nomogram models to predict radiotherapy or chemotherapy benefit in stage III/IV gastric adenocarcinoma with surgery

**DOI:** 10.3389/fonc.2023.1223857

**Published:** 2023-08-14

**Authors:** Xiangqing Ren, Tian Huang, Xiaolong Tang, Qian Ma, Ya Zheng, Zenan Hu, Yuping Wang, Yongning Zhou

**Affiliations:** ^1^ The First Clinical Medical College, Lanzhou University, Lanzhou, China; ^2^ Department of Gastroenterology, the First Hospital of Lanzhou University, Lanzhou, China; ^3^ Key Laboratory for Gastrointestinal Diseases of Gansu Province, The First Hospital of Lanzhou University, Lanzhou, China; ^4^ Geriatrics Department, Xianyang First People’s Hospital, Xianyang, China

**Keywords:** gastric cancer, chemotherapy, radiotherapy, SEER, prognostic model, nomogram

## Abstract

**Objectives:**

The advanced gastric adenocarcinoma (GAC) patients (stage III/IV) with surgery may have inconsistent prognoses due to different demographic and clinicopathological factors. In this retrospective study, we developed clinical prediction models for estimating the overall survival (OS) and cancer-specific survival (CSS) in advanced GAC patients with surgery

**Methods:**

A retrospective analysis was conducted using the Surveillance, Epidemiology, and End Results (SEER) database. The total population from 2004 to 2015 was divided into four levels according to age, of which 179 were younger than 45 years old, 695 were 45-59 years old, 1064 were 60-74 years old, and 708 were older than 75 years old. There were 1,712 men and 934 women. Univariate and multivariate Cox regression analyses were performed to identify prognostic factors for OS and CSS. Nomograms were constructed to predict the 1-, 3-, and 5-year OS and CSS. The models’ calibration and discrimination efficiency were validated. Discrimination and accuracy were evaluated using the consistency index, area under the receiver operating characteristic curve, and calibration plots; and clinical usefulness was assessed using decision curve analysis. Cross-validation was also conducted to evaluate the accuracy and stability of the models. Prognostic factors identified by Cox regression were analyzed using Kaplan-Meier survival analysis.

**Results:**

A total of 2,646 patients were included in our OS study. Age, primary site, differentiation grade, AJCC 6^th^_TNM stage, chemotherapy, radiotherapy, and number of regional nodes examined were identified as prognostic factors for OS in advanced GAC patients with surgery (*P* < 0.05). A total of 2,369 patients were included in our CSS study. Age, primary site, differentiation grade, AJCC 6^th^_TNM stage, chemotherapy, radiotherapy, and number of regional nodes examined were identified as risk factors for CSS in these patients (*P* < 0.05). These factors were used to construct the nomogram to predict the 1-, 3-, and 5-year OS and CSS of advanced GAC patients with surgery. The consistency index and area under the receiver operating characteristic curve demonstrated that the models effectively differentiated between events and nonevents. The calibration plots for 1-, 3-, and 5-year OS and CSS probability showed good consistence between the predicted and the actual events. The decision curve analysis indicated that the nomogram had higher clinical predictive value and more significant net gain than AJCC 6^th^_TNM stage in predicting OS and CSS of advanced GAC patients with surgery. Cross-validation also revealed good accuracy and stability of the models.

**Conclusion:**

The developed predictive models provided available prognostic estimates for advanced GAC patients with surgery. Our findings suggested that both OS and CSS can benefit from chemotherapy or radiotherapy in these patients.

## 1 Introduction

Gastric cancer (GC) is a common malignancy and the fourth leading cause of cancer-related deaths, which places a heavy burden on public healthcare (1, 2). Among all types of cancer, GC incidence ranks fourth in men and seventh in women (1, 3). The most common histopathologic subtype of GC is gastric adenocarcinoma (GAC) (4). Many GAC patients are initially diagnosed with advanced stage III/IV cancer, especially in developing countries (5). Currently, surgery remains the only curable treatment. However, even after complete excision, local recurrence rates remain high (6). Therefore, clinicians are interested in adjuvant chemotherapy (CT) or radiotherapy (RT) (7). Because the stomach is a peristaltic cavity, few stage III/IV GAC patients receive standard chemoradiotherapy (CRT) throughout treatment after surgical resection (8). Additionally, few large-scale prospective clinical trials have been attempted, making it difficult for clinicians to determine whether adjuvant therapy can benefit patients (9–11). Predictive models can provide some insights into these essential clinical issues.

Common clinical CT drugs such as cisplatin and fluorouracil are known to have an excellent inhibitory effect on GAC, but significant side effects exist (12). In addition, the application of RT in GAC remains limited and the standard use of RT remains controversial (13). Currently, there is no consensus on whether stage III/IV GAC patients undergoing surgery benefit from adjuvant CT or adjuvant RT and to what extent (14, 15).

Several studies have established nomogram models based on the SEER databases to personalize predictions of benefits of adjuvant CT or RT in GAC patients (16–22). However, our study population and models design scheme were different from those of previous studies, as we did not examine the effects of surgery, adjuvant CT, and RT alone. We are aware that surgery remains the preferred treatment choice for stage III/IV GA patients. Therefore, our research focused on whether and to what extent stage III/IV GA patients receiving surgery benefit from adjuvant CT or adjuvant RT. Through these analyses, we aimed to assess whether GAC patients derive benefits from adjuvant CT or adjuvant RT. Additionally, we developed nomogram models to predict the extent of benefits from adjuvant CT or adjuvant RT.

## 2 Materials and methods

### 2.1 Data extraction

For this retrospective study, we used the SEER database, a population-based reporting system, covers nearly 50% of the U.S. population. We accessed the clinical features of GAC patients from the National Cancer Institute’s SEER*Stat software (Version 8.3.2). Ethical consent was waived due to the SEER database contains anonymous patient information.

### 2.2 Patients

The inclusion criteria of the study were as follows:

1. Age > 18 years;
2. Accurate information on race and sex;
3. GAC diagnosis;
4. Accurate differentiation grade;
5. Accurate pathological diagnosis;
6. Accurate staging information for AJCC 6^th^_T, AJCC 6^th^_ N, and AJCC 6^th^_M stages;
7. AJCC 6^th^_Stage III or IV;
8. Clear history of surgery;
9. Recorded number of regional nodes examined;
10. Accurate information on follow-up time and outcomes.

We excluded patients with zero survival time, as well as those who survived less than 1 month. Patients with unknown information regarding CT or RT were included in the group without CT or RT.

### 2.3 Clinical characteristic and outcome variables

We categorized the patients’ age according to the international age classification standard. The total population from 2004 to 2015 was divided into four levels, of which 179 were younger than 45 years old, 695 were 45-59 years old, 1064 were 60-74 years old, and 708 were older than 75 years old (23, 24). There were 1,712 men and 934 women. The outcomes of interest in this study were overall survival (OS) and cancer-specific survival (CSS). OS is the time from diagnosis or treatment until death from any cause. CSS is the time from diagnosis or treatment until death specifically from the cancer, and deaths from other causes are not included. These two endpoints provide different information and can be used to understand the effectiveness of a treatment or the progression of a disease.

### 2.4 Statistical analysis

Univariate and multivariate Cox regression were conducted to identify factors related to OS and CSS, and the risk ratio (HR) and 95% confidence interval (95% CI) were calculated to evaluate the impact of relevant clinical indicators on the prognosis of patients. Based on those analyses, nomogram models were created to predict the 1-, 3-, and 5-year OS and CSS of advanced GAC patients with surgery. In addition, we used the Kaplan-Meier estimation and log-rank test to assess the association between the variables and the OS/CSS. The performance of the nomogram models was evaluated using the C-index and area under the curve (AUC) of the ROC curves to assess their ability to distinguish between events and nonevents. Calibration plots were created for 1-, 3-, and 5-year OS and CSS probabilities to compare the predicted and actual events. Additionally, DCA was conducted to compare the decisional net benefit of the models with AJCC 6^th^_TNM stage. The Cross-validation was also used to evaluate the accuracy and stability of the models. *P* < 0.05 was considered statistically significant. The research was performed using python (PSF, version 3.7) and R software (RA, version 3.6.3).

## 3 Results

### 3.1 Patient demographic and clinicopathological characteristics

**Figure 1:**
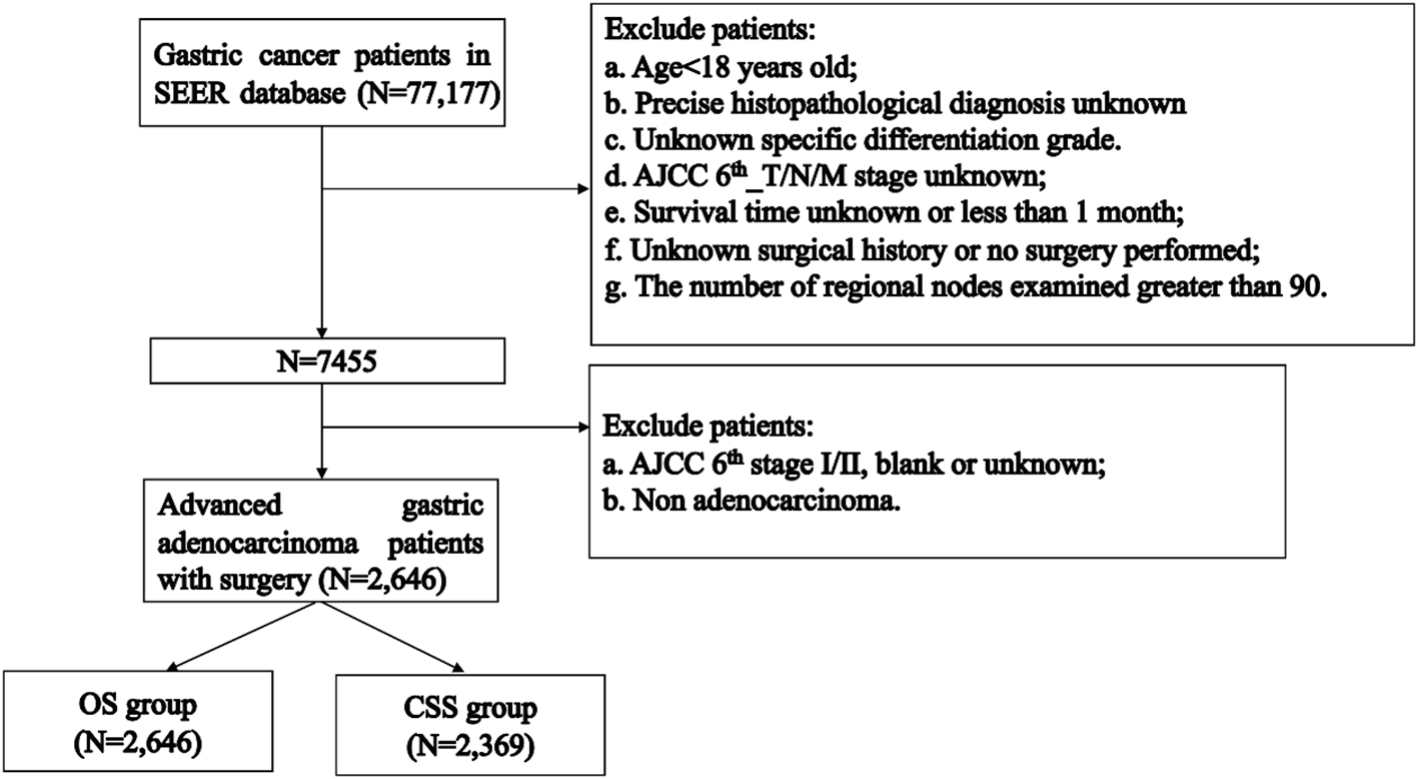
Flowchart of participants inclusion and exclusion.

The flowchart of patients selecting was shown in **Figure 1**. We extracted demographic and clinical data of 77,177 GC patients from the SEER database. After applying our inclusion criteria, a total of 2,646 patients were finally included in our OS study of advanced GAC patients with surgery. **Table 1** presents detailed demographic and clinical characteristics of advanced GAC patients with surgery from 2004 to 2015. Age was divided into four groups, with the largest proportion of patients falling in the 60-74 years age range (40.2%). Among the patients, 63.3% belonged to the white population, 64.1% were married, and 64.7% were males. Cardiac/fondus of the stomach was the most common primary site of the disease, accounting for 31.6%. The majority of patients (73.7%) had Grade III cancer based on pathological differentiation. In terms of the AJCC 6^th^_T/N/M stages, T3 accounted for 51.4%; N1, 41.3%; and M0, 76.5% of the patients. Of the total patients, 69.2% received CT, while 45.6% received RT. The majority of patients (27.6%) had 1-11 regional nodes examined.

**Table 1:** Univariate and multivariate Cox regression analysis of OS.

Variables	OS group (n=2646)	Univariate analysis			Multivariate analysis		
		HR	95%CI	*P*	HR	95%CI	*P*
Age, n (%)							
**<45**	179 (6.8)	**Ref**					
**45-59**	695 (26.3)	0.908	0.756,1.092	0.305	0.994	0.825, 1.198	0.951
**60-74**	1064 (40.2)	1.038	0.870,1.237	0.682	1.137	0.950, 1.362	0.162
**75+**	708 (26.8)	1.560	1.302,1.868	**0**	1.471	1.216, 1.778	**0**
Race, n (%)							
**Others**	627 (23.7)	**Ref**					
**Black**	345 (13.0)	1.139	0.985,1.317	0.079			
**White**	1674 (63.3)	1.091	0.984,1.209	0.099			
Marital, n (%)							
**Others**	949 (35.9)	**Ref**					
**Married**	1697 (64.1)	0.832	0.762,0.908	**0**	0.918	0.838, 1.006	0.068
Gender, n (%)							
**Female**	934 (35.3)	**Ref**					
**Male**	1712 (64.7)	0.891	0.816,0.973	**0.010**	0.979	0.890, 1.077	0.660
Primary site, n (%)							
**Cardia/Fundus of stomach**	835 (31.6)	**Ref**					
**Body/Lesser curvature/Greater curvature of stomach**	552 (20.9)	1.154	1.023,1.300	**0.019**	0.799	0.703, 0.908	**0.001**
**Antrum/Pylorus**	763 (28.8)	1.206	1.081,1.345	**0.001**	0.881	0.784, 0.990	**0.033**
**Others**	496 (18.7)	1.335	1.180,1.510	**0**	0.924	0.808, 1.056	0.245
Differentiation Grade, n (%)							
**I**	61 (2.3)	**Ref**					
**II**	557 (21.1)	1.242	0.914,1.689	0.166	1.206	0.886, 1.643	0.234
**III**	1949 (73.7)	1.561	1.161,2.100	**0.003**	1.546	1.145, 2.088	**0.004**
**IV**	79 (3.0)	1.490	1.017,2.182	**0.041**	1.438	0.976, 2.119	0.066
AJCC 6th_T, n (%)							
**T1**	26 (0.1)	**Ref**					
**T2**	752 (28.4)	1.447	0.894,2.344	0.133	1.701	1.041, 2.778	**0.034**
**T3**	1360 (51.4)	1.477	0.915,2.386	0.111	2.279	1.400, 3.712	**0.001**
**T4**	508 (19.2)	1.730	1.065,2.809	**0.027**	2.696	1.648, 4.409	**0**
AJCC 6th _N, n (%)							
**N0**	131 (5.0)	**Ref**					
**N1**	1094 (41.3)	1.294	1.042,1.606	**0.020**	1.858	1.481, 2.329	**0**
**N2**	1008 (38.1)	1.522	1.226,1.890	**0**	2.949	2.335, 3.726	**0**
**N3**	413 (15.6)	2.197	1.747,2.764	**0**	4.64	3.600, 5.980	**0**
AJCC 6th _M, n (%)							
**M0**	2023 (76.5)	**Ref**					
**M1**	623 (23.5)	1.793	1.628,1.975	**0**	1.937	1.743, 2.152	**0**
Radiotherapy, n (%)							
**No/unknown**	1440 (54.4)	**Ref**					
**Yes**	1206 (45.6)	0.568	0.521,0.619	**0**	0.862	0.774, 0.961	**0.007**
Chemotherapy, n (%)							
**No/unknown**	814 (30.8)	**Ref**					
**Yes**	1832 (69.2)	0.485	0.444,0.530	**0**	0.564	0.504, 0.631	**0**
Regional nodes examined, n (%)							
**1-11**	730 (27.6)	**Ref**					
**12-17**	611 (23.1)	0.892	0.794,1.002	0.055	0.860	0.761, 0.972	**0.016**
**18-25**	687 (26.0_	0.792	0.706,0.889	**0**	0.667	0.589, 0.756	**0**
**26-90**	618 (23.4)	0.728	0.646,0.820	**0**	0.554	0.482, 0.636	**0**

"Ref" in boldface is a contrast, indicating "emphasis"; "Numbers in bold" are indicative of a "statistical difference".

The CSS study included 2,369 patients. **Table 2** presents demographic and clinical characteristics of the CSS cohort.

**Table 2:** Univariate and multivariate Cox regression analysis of CSS.

Variables	CSS group (n=2369)	Univariate analysis			Multivariate analysis		
		HR	95%CI	*P*	HR	95%CI	*P*
Age, n (%)							
**<45**	166 (7.0)	**Ref**					
**45-59**	646 (27.3)	0.913	0.753,1.106	0.352	0.97	0.798, 1.179	0.759
**60-74**	953 (40.2)	1.022	0.849,1.230	0.816	1.103	0.913, 1.334	0.310
**75+**	604 (25.5)	1.620	1.338,1.960	**0**	1.499	1.228, 1.831	**0**
Race, n (%)							
**Others**	561 (23.7)	**Ref**					
**Black**	301 (12.7)	1.131	0.966,1.323	0.126			
**White**	1507 (63.6)	1.113	0.997,1.244	0.057			
Marital, n (%)							
**Others**	829 (35.0)	**Ref**					
**Married**	1540 (65.0)	0.843	0.767,0.926	**0**	0.929	0.841, 1.026	0.145
Gender, n (%)							
**Female**	855 (36.1)	**Ref**					
**Male**	1514 (63.9)	0.886	0.807,0.973	**0.011**	0.969	0.875, 1.073	0.548
Primary site, n (%)							
**Cardia/Fundus of stomach**	760 (32.1)	**Ref**					
**Body/Lesser curvature/Greater curvature of stomach**	493 (20.8)	1.180	1.038,1.341	**0.011**	0.813	0.710, 0.932	**0.003**
**Antrum/Pylorus**	667 (28.2)	1.226	1.090,1.379	**0.001**	0.877	0.774, 0.995	**0.041**
**Others**	449 (19.0)	1.339	1.175,1.527	**0**	0.882	0.765, 1.018	0.087
Differentiation Grade, n (%)							
**I**	51 (2.2)	**Ref**					
**II**	488 (18.9)	1.324	0.936,1.873	0.113	1.349	0.951, 1.914	0.093
**III**	1758 (74.2)	1.715	1.226,2.399	**0.002**	1.772	1.261, 2.490	**0.001**
**IV**	72 (3.0)	1.646	1.081,2.508	**0.020**	1.623	1.058, 2.492	**0.027**
AJCC 6th_T, n (%)							
**T1**	22 (0.9)	**Ref**					
**T2**	681 (28.7)	1.628	0.939,2.822	0.083	1.906	1.091, 3.332	**0.024**
**T3**	1220 (51.5)	1.637	0.947,2.830	0.078	2.537	1.456, 4.421	**0.001**
**T4**	446 (18.8)	2.030	1.167,3.531	**0.012**	3.166	1.809, 5.541	**0**
AJCC 6th _N, n (%)							
**N0**	111 (4.7)	**Ref**					
**N1**	963 (40.7)	1.260	0.987,1.607	0.064	1.76	1.364, 2.272	**0**
**N2**	915 (38.6)	1.522	1.193,1.942	**0.001**	2.893	2.228, 3.758	**0**
**N3**	380 (16.0)	2.227	1.724,2.877	**0**	4.402	3.328, 5.822	**0**
AJCC 6th _M, n (%)							
**M0**	1793 (75.7)	**Ref**					
**M1**	576 (24.3)	1.840	1.662,2.038	**0**	1.925	1.722, 2.151	**0**
Radiotherapy, n (%)							
**No/unknown**	1276 (53.9)	**Ref**					
**Yes**	1093 (46.1)	0.556	0.507,0.610	**0**	0.849	0.756, 0.953	**0.006**
Chemotherapy, n (%)							
**No/unknown**	687 (29.0)	**Ref**					
**Yes**	1682 (71.0)	0.461	0.419,0.508	**0**	0.554	0.492, 0.625	**0**
Regional nodes examined, n (%)							
**1-11**	626 (26.4)	**Ref**					
**12-18**	650 (27.4)	0.845	0.749,0.954	0.007	0.783	0.689, 0.891	**0**
**18-25**	532 (22.5)	0.812	0.715,0.923	0.001	0.663	0.576, 0.763	**0**
**26-90**	561 (23.7)	0.723	0.635,0.822	**0**	0.538	0.463, 0.625	**0**

"Ref" in boldface is a contrast, indicating "emphasis"; "Numbers in bold" are indicative of a "statistical difference".

### 3.2 Identification of independent prognostic factors

In the OS group of advanced GAC patients with surgery, the results of univariate Cox regression analysis (**Table 1**) revealed that eleven factors (age, marital status, gender, primary site, differentiation grade, AJCC 6^th^_T/N/M stages, CT, RT, and number of regional nodes examined) were significantly associated with patients’ OS (*P* < 0.05). Subsequently, multivariate Cox regression analysis was conducted using these eleven factors. Nine variables, namely age, primary site, differentiation grade, AJCC 6^th^_T/N/M stages, CT, RT, and number of regional nodes examined, were identified for constructing the OS nomogram.

For CSS analysis in the advanced GAC patients with surgery (**Table 2**), the univariate Cox regression analysis revealed that eleven factors (age, marital status, gender, primary site, differentiation grade, AJCC 6^th^_T/N/M stages, CT, RT, and number of regional nodes examined) were significantly associated with CSS (*P* < 0.05). Subsequently, multivariate Cox regression analysis was conducted using these eleven factors. Nine variables, namely age, primary site, differentiation grade, AJCC 6^th^_T/N/M stages, CT, RT, and number of regional nodes examined, were identified for constructing the CSS nomogram.

### 3.3 Construction of the prognostic nomogram models

Based on the independent survival prognostic factors determined by Cox regression analysis, we constructed the OS and CSS nomograms for advanced GAC patients with surgery.

**Figure 2:**
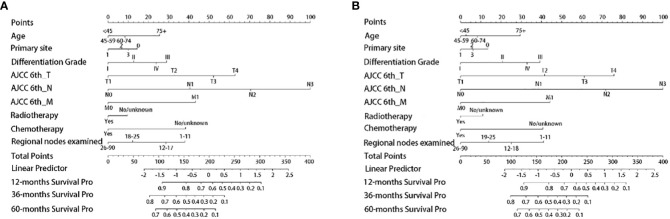
Establishment of nomogram models. **(A)** The nomogram model of OS; **(B)** the nomogram model of CSS. Primary site: 0, Cardia/Fundus of stomach; 1, Body/Lesser curvature/Greater curvature of stomach; 2, Antrum/Pylorus; 3, Others.

According to both the OS (**Figure 2A**) and CSS (**Figure 2B**) nomograms, AJCC 6^th^_N stage contributed the most to the OS and CSS, respectively, followed by AJCC 6^th^_T stage, AJCC 6^th^_M stage, regional nodes examined, CT, differentiation grade, age, primary site, and RT.

### 3.4 Validation of the nomograms

**Figure 3:**
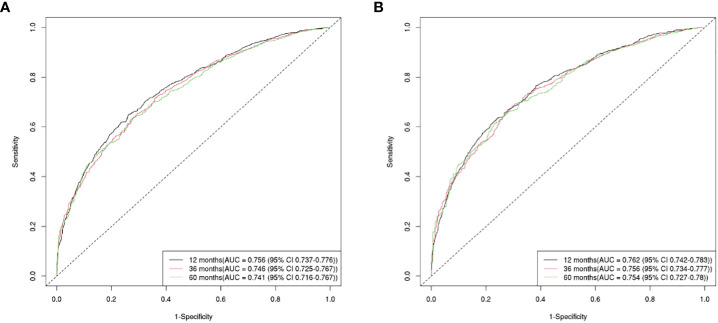
The AUC value of the ROC curve for 1, 3, and 5-year. **(A)** The 1, 3, and 5-year AUC value of OS nomogram; **(B)** the 1, 3, and 5-year AUC value of CSS nomogram.

**Figure 4:**
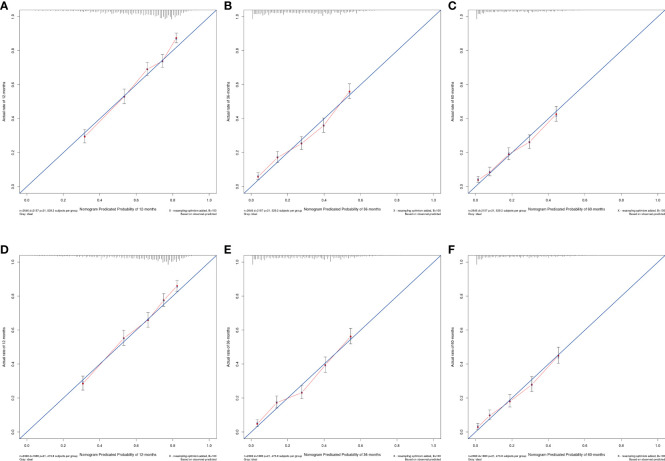
Calibration plots of nomogram models. **(A–C)** the 1, 3, and 5-year calibration plot of OS, respectively; **(D–F)** the 1, 3, and 5-year calibration plot of CSS, respectively.

**Figure 5:**
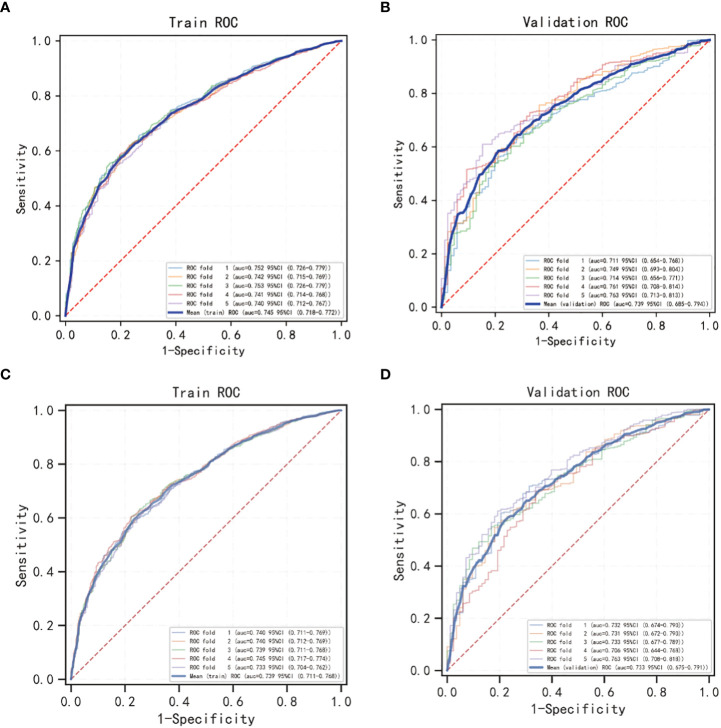
5-fold cross validation of OS and CSS nomogram models using Logistic regression classification machine learning models. **(A, B)** The cross validation of the OS model; **(C, D)** the cross validation of the CSS model.

The OS nomogram validation results were as follows: the C-index value was 0.685 (0.673, 0.697) and the AUC value of the ROC curve for 1-, 3-, and 5 years was 0.756, 0.746, and 0.741, respectively (**Figure 3A**). The calibration chart of the OS nomogram revealed high consistency between the predicted and actual data (**Figures 4A–C**). Cross-validation also revealed good accuracy and stability of the models (**Figures 5A, B**).

The CSS nomogram validation results were as follows: the C-index value was 0.691 (0.679, 0.704), and the AUC value of the ROC curve for 1-, 3-, and 5 years was 0.762, 0.756, and 0.754, respectively (**Figure 3B**). The calibration chart of the CSS nomogram revealed high consistency between the predicted and actual data (**Figures 4D–F**). Cross-validation also demonstrated good accuracy and stability of the models (**Figures 5C, D**).

### 3.5 Nomogram models comparison with AJCC 6^th^_TNM stage

**Figure 6:**
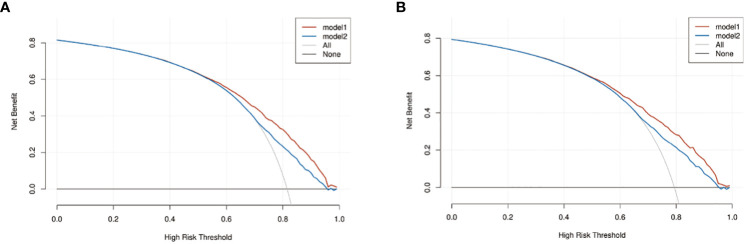
The decision curve analysis (DCA) of OS and CSS nomograms. **(A)** The DCA of OS nomogram; **(B)** the DCA of CSS nomogram. Model1: nomogram model; model2: AJCC 6^th^_TNM.

We compared the nomogram models with the AJCC 6^th^_TNM stage. DCA was used to assess the models’ clinical feasibility. The nomogram showed better clinical predictive value and yielded a higher net gain in predicting OS and CSS than the AJCC 6^th^_TNM stage (**Figures 6A, B**).

### 3.6 Kaplan-Meier survival analysis

**Figure 7:**
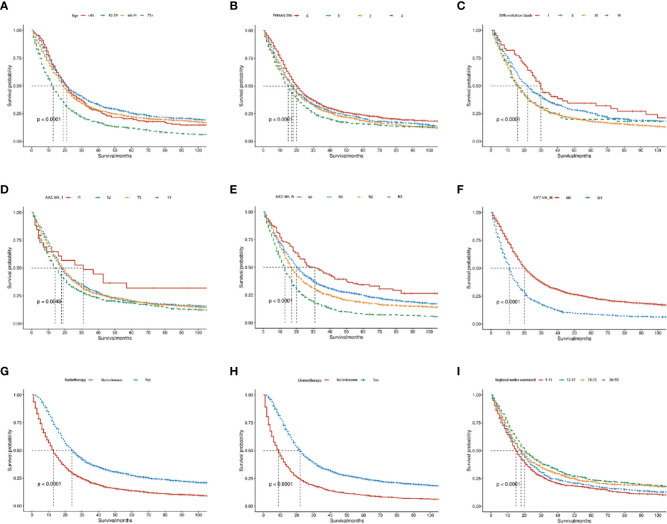
The Kaplan–Meier curves of OS in advanced GAC patients with surgery. **(A–I)** Kaplan–Meier curves for OS in the age, primary site, differentiation grade, AJCC 6^th^_T, AJCC 6^th^_N, AJCC 6^th^_M, radiotherapy, chemotherapy and regional nodes examined, separately. *P*<0.05 was statistically significant.

The Kaplan-Meier survival analysis of the OS and CSS group showed that middle-aged patients (45-59 years) had the best prognosis, whereas patients aged >75 years had the worst prognosis (*P* < 0.05, **Figure 7A**; **Supplementary Figure 1A**). As for primary site, cardia and fundus lesion had the worst prognosis (*P* < 0.05, **Figure 7B**; **Supplementary Figure 1B**). Regarding the differentiation grade, grade III/IV had the worst prognosis (*P* < 0.05, **Figure 7C**; **Supplementary Figure 1C**). Regarding the AJCC 6^th^_ T stage, the prognosis of T1 stage patients was significantly better (*P* < 0.05, **Figure 7D**; **Supplementary Figure 1D**). Regarding the AJCC 6^th^_ N stage, the prognosis of N0 stage patients was significantly better (*P* < 0.05, **Figure 7E**; **Supplementary Figure 1E**). Regarding the AJCC 6^th^_ M stage, the prognosis of M0 stage patients was significantly better (*P* < 0.05, **Figure 7F**; **Supplementary Figure 1F**). Additionally, patients who received RT or CT had a better prognosis (*P* < 0.05, **Figures 7G, H**; **Supplementary Figures 1G, H**). Moreover, the prognosis of patients with 26-90 regional nodes examined was better (*P* < 0.05, **Figure 7I**; **Supplementary Figure 1I**).

## 4 Discussion

In this study, we analyzed the clinical information of 2,646 advanced GAC patients with surgery and developed prognostic models through univariate and multivariate Cox regression analyses. AJCC 6th_T/N/M stages, regional nodes examined, CT, differentiation grade, age, primary site, and RT significantly affected those patients’ OS and CSS.

Our nomogram models showed that both CT and RT were independently correlated with OS and CSS in advanced GAC patients who underwent surgery, while surgical excision remains the primary treatment option, a multidisciplinary approach is standard for advanced resectable GAC (25). Adjuvant CT after D2 gastrectomy is the standard regimen for resectable locally advanced GAC in Asia (26). Currently, S-1 monotherapy, one of the most common regimens, has been questioned for its lack of poor efficacy in preventing the disease from worsening and inability to reduce the hematogenic recurrence (27, 28). In Europe, perioperative CT with FLOT regimen (5-fluorouracil, folinic acid, oxaliplatin, and docetaxel) is the standard treatment (29, 30). For patients with advanced GAC, adjuvant CT is recommended for postoperative routine treatment, but not all patients benefit from CT, and some may even be harmed (29, 31–33). The survival benefits of CT in early GAC patients are poorly understood, as some studies have not reported a significant improvement in survival (34, 35). Therefore, further clinical studies are needed to determine which subgroup of GAC patients benefit more from CT and to what extent.

Based on our results, advanced GAC patients can benefit from RT; however, unfortunately, it did not achieve the expected clinical effect. In a phase III study by Zhu et al. (36), adjuvant CRT was reported to benefit patients with D2 gastrectomy. The trial enrolled 380 D2 gastrectomy patients who were divided into intensity-modulated radiation therapy (IMRT) plus CT and CT alone. After a follow-up of no less than 5 years, IMRT plus CT resulted in significantly improved disease-free survival (DFS) and reduced local recurrence among patients with positive nodes, despite no difference in OS. Most trials comparing CRT and CT in GAC patients were not prospective studies and had small sample sizes (37, 38). Lee et al. (14) conducted a GAC adjuvant CRT trial, randomly dividing 458 patients into the CT and CRT groups. After 53 months of follow-up, they observed no significant difference in DFS between the groups at 3 years, while a positive effect of RT and CT on tumor recurrence. Notably, the 3-year DFS for CRT was significantly longer than that for CT in patients with positive lymph nodes. GAC bleeding reduces a patient’s quality of life and can be life-threatening due to hematological instability. Multiple clinical studies have shown that RT is an effective and well-tolerated method for controlling GAC bleeding (39–41), particularly in patients with poor performance or inoperable advanced stage. Thus, the efficacy of RT for GAC remains controversial and requires confirmation through large-scale clinical data.

Our results also found that primary site, differentiation grade, T/N/M/ stages, and lymph node examination significantly affected patient survival. Our study showed that patients with cardiac cancer had a worse survival benefit, regardless of OS or CSS. A multicenter study from China found that compared with non-cardiac GC, patients with cardiac GC had a significantly higher proportion of males, were older, had a more advanced pathological stage, and had poorer clinicopathological features at diagnosis. Therefore, the 5-year survival rate in the cardiac GC group was significantly lower than that in the non-cardiac GC group (42). A cohort study from 1987-1991 to 2012-2016 found that among non-cardia adenocarcinoma patients who underwent surgery, five-year survival increased from 29% to 38%, while survival increased from 4% to 7% for those who underwent CT; In cardiac adenocarcinoma, five-year survival increased from 16% to 40% for patients undergoing surgery and from 0% to 5% for those undergoing CT (43). As can be seen from the nomogram, the low differentiation grades of the GAC were significantly associated with poorer survival, which was consistent with many previous studies. Lu et al. (44) found that the tumor differentiation grade, body mass index, ascites, and CT were independent prognostic factors for advanced or metastatic GC in elderly patients. A clinical study from the SEER database also found that the poor pathologic differentiation grade, cardiac cancer, white race, young, and higher N stage were positively correlated with bone metastasis (45).

At present, the TNM classification of GC has been widely accepted worldwide (46). Our nomogram models showed that the T/N/M stages were critical prognostic factors for advanced GAC. Zhong et al. (47) found that TNM staging and radical surgery were independent prognostic factors affecting OS in young GC patients. Sun et al. (48) explored various factors affecting the survival of elderly patients with locally advanced GC. Statistical analysis showed that the T/N/M stages were independent prognostic factors for OS and CSS. Based on the multivariate cox regression analysis and nomogram models in our study, the number of regional nodes examined was shown to be a protective factor for OS and CSS in GAC patients. Lin et al. (49) reported that GAC patients’ OS can be improved by examining more regional nodes, depending on reducing the lymph node noncompliance rate. Similarly, Macalindong et al. (50) concluded that larger lymph node harvest can significantly improve DFS and OS in GAC patients.

However, there are some limitations to this study. First, our original data is collected from the SEER database, which collected cancer patient information from multiple regions and hospitals in the United States, leading to potential differences in pathological evaluation criteria and treatment regimens. Second, the SEER database lacks data such as specific CT drugs and regimens, RT courses, and the sequence of RT and CT before and after surgery, which are crucial for stage III/IV GAC patients. Finally, although we performed a reliable validation, the efficacy of this validation was not ideal because both the training group and the validation group were from the SEER database. Therefore, the validation of larger prospective clinical trials is warranted.

## 5 Conclusion

In conclusion, we developed nomogram models to predict the OS and CSS of the advanced GAC patients with surgery, and found that they can benefit from adjuvant CT or RT.The models serve as a valuable tool for clinicians and patients in quantifying the potential benefits of adjuvant CT or RT, providing guidance for the treatment of advanced GAC.

## Data availability statement

The original contributions presented in the study are included in the article/**Supplementary Material**. Further inquiries can be directed to the corresponding authors.

## Ethics statement

Ethical consent was waived due to the SEER database contains anonymous patient information.

## Author contributions

Conceptualization: XR, TH. Methodology: XR, TH, XT. Writing—original draft: TH, XT. Data processing: TH, YaZ, XT. Validation: ZH, YW, QM. Supervision: YoZ, YW. Writing—review & editing: TH, ZH, YoZ. All authors contributed to the article and approved the submitted version.
