# Supplementary material for: Development and validation of nomogram models to predict radiotherapy or chemotherapy benefit in stage III/IV gastric adenocarcinoma with surgery
Source: Front Oncol. 2023 Aug 14;13:1223857. doi: 10.3389/fonc.2023.1223857 (PMC10466399; doi:10.3389/fonc.2023.1223857)
Supplement: Supplementary file 1 [file DataSheet_1.docx]

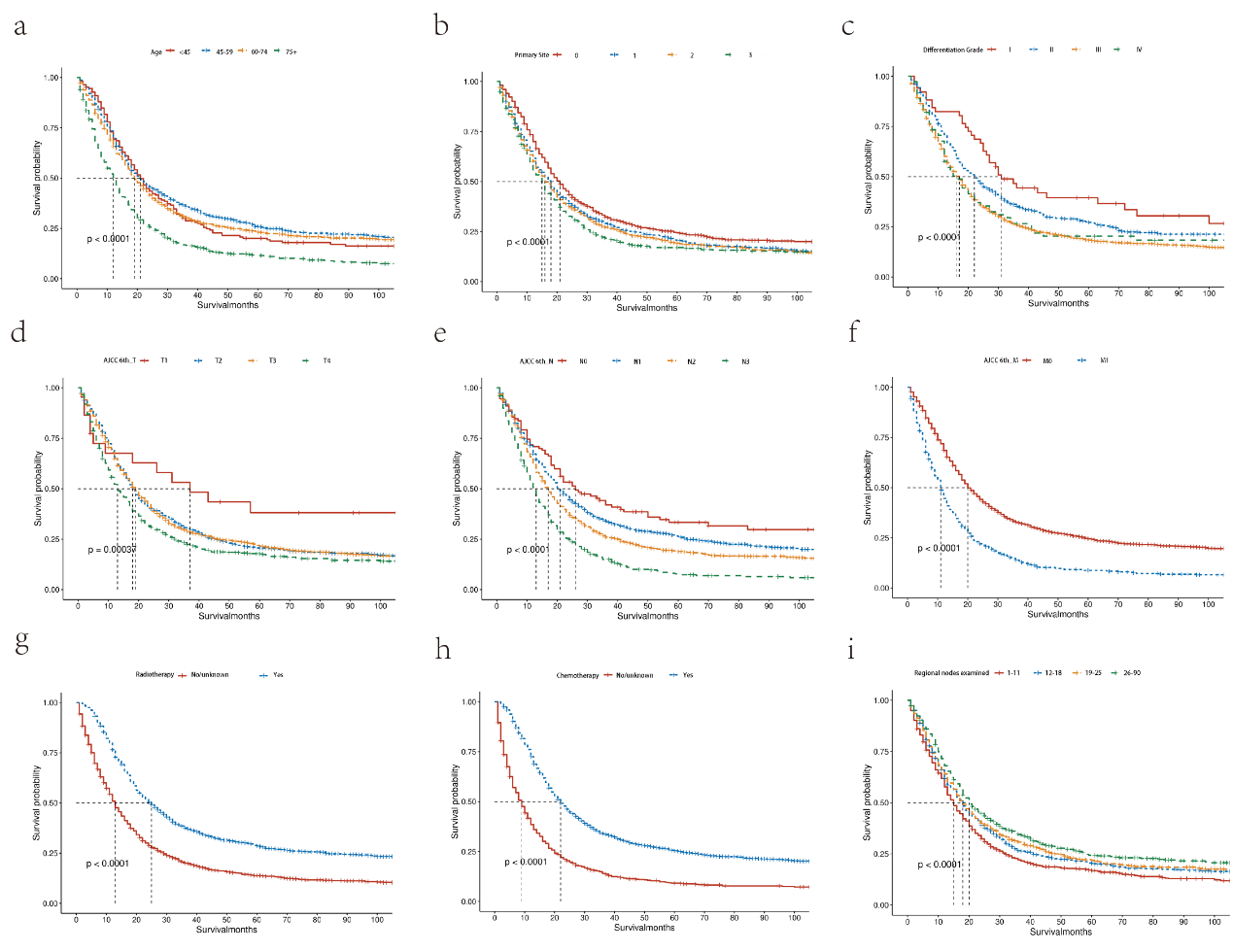


Primary site: 0, Cardia/Fundus of stomach; 1, Body/Lesser curvature/Greater curvature of stomach; 2, Antrum/Pylorus; 3, Others.

**Supplementary Figure 1** The Kaplan–Meier curves of CSS in advanced GAC patients with surgery. a, b, c, d, e, f, g, h and i: Kaplan–Meier curves for CSS in the age, primary site, differentiation grade, AJCC 6^th^_T, AJCC 6^th^_N, AJCC 6^th^_M, radiotherapy, chemotherapy and regional nodes examined, separately. *P*<0.05 was statistically significant.
